# Inhibition of the TRAIL Death Receptor by CMV Reveals Its Importance in NK Cell-Mediated Antiviral Defense

**DOI:** 10.1371/journal.ppat.1004268

**Published:** 2014-08-14

**Authors:** Shilpi Verma, Andrea Loewendorf, Qiao Wang, Bryan McDonald, Alec Redwood, Chris A. Benedict

**Affiliations:** 1 Division of Immune Regulation, La Jolla Institute for Allergy and Immunology, La Jolla, California, United States of America; 2 Microbiology and Immunology, School of Pathology and Laboratory Medicine, University of Western Australia, Crawley, Western Australia, Australia; University of California, Berkeley, United States of America

## Abstract

TNF-related apoptosis inducing ligand (TRAIL) death receptors (DR) regulate apoptosis and inflammation, but their role in antiviral defense is poorly understood. Cytomegaloviruses (CMV) encode many immune-modulatory genes that shape host immunity, and they utilize multiple strategies to target the TNF-family cytokines. Here we show that the m166 open reading frame (orf) of mouse CMV (MCMV) is strictly required to inhibit expression of TRAIL-DR in infected cells. An MCMV mutant lacking m166 expression (m166^stop^) is severely compromised for replication *in vivo*, most notably in the liver, and depleting natural killer (NK) cells, or infecting TRAIL-DR^−/−^ mice, restored MCMV-m166^stop^ replication completely. These results highlight the critical importance for CMV to have evolved a strategy to inhibit TRAIL-DR signaling to thwart NK-mediated defenses.

## Author Summary

TRAIL death receptors regulate apoptosis and inflammation, and growing evidence suggests their importance in promoting antiviral defenses. Many viruses encode strategies to modulate signaling by TNF family cytokines in order to shape host defenses. Cytomegaloviruses encode many immune modulatory genes, many of which target the TNF family, highlighting their critical role in host antiviral immunity. Here we show that the mouse cytomegalovirus (MCMV) m166 protein restricts cell surface expression of the TRAIL death receptor in infected cells, thus protecting them from TRAIL mediated apoptosis. An MCMV mutant lacking m166 gene expression (MCMV-m166^stop^) is severely attenuated for replication *in vivo*, especially in the liver, where a population of immature natural killer (NK) cells resides that express very high levels of TRAIL. These TRAIL-expressing NK cells are critical for antiviral defense, as their depletion restores replication of MCMV-m166^stop^ to wildtype levels. In addition, replication of MCMV-m166^stop^ is normal in TRAIL-DR deficient mice, definitively demonstrating the importance for m166-mediated inhibition of this TNFR in promoting viral replication and subverting host innate defenses.

## Introduction

Tumor necrosis factor (TNF)-family cytokines regulate multiple aspects of host antiviral immunity, in part through their abilities to promote cell death and survival [1]. In many cases, this involves both the direct killing of virally infected cells, as well as assisting in terminating the host effector response after infection is controlled in order to limit tissue damage and restore homeostasis. Herpesviruses induce robust host defenses upon infection, including many mediated by TNF-family cytokines, but nevertheless successfully establish a lifelong infection. This is due in large part because they encode an enormous number of immune-modulatory proteins, a fact epitomized by cytomegalovirus (CMV, the prototypic β-herpesvirus) [2], [3], the largest of the human herpesviruses that expresses >700 protein-encoding transcripts [4] derived from its >230 kb genome. As CMV, as well as other herpesviruses, encode multiple strategies targeting the TNF-family [1], [5], this strongly suggests their importance in defense to these persistent viruses and the necessity for the evolution of viral counterstrategies targeting them.

Human CMV (HCMV/HHV5) infects the majority of people worldwide, with incidence increasing with age and varying by race, geography and socioeconomic standing [6]. Primary infection is usually asymptomatic in healthy individuals, however in people with suppressed or naive immune systems it can result in serious disease, and the high incidence of congenital infection is a driving force for vaccine development [7]. Natural killer (NK) cells are critical for controlling HCMV infection and disease [8], [9], and recent data indicate the virus preferentially impacts NK subsets expressing the NKG2C activating receptor [10], [11]. In turn, HCMV utilizes multiple strategies to dampen NK-mediated defenses in order to promote its replication and dissemination [12]. As CMV replication is highly species specific, mouse CMV (MCMV; a natural mouse pathogen) has proved to be an invaluable model for studying CMV infection and immunity. MCMV has provided many key insights into mechanisms of NK-mediated antiviral immunity through both direct recognition of infected cells (e.g. m157-Ly49H interaction) [13], [14] and cytokine-induced activation (e.g. IL-12 and type I interferon (IFN-I)) [15].

TNF-related apoptosis inducing ligand (TRAIL/TNFSF10) binds multiple receptors in humans and mice, including both death (DR) and ‘decoy’ receptors [16], [17]. While humans encode two DRs for TRAIL (TRAIL-R1/DR4 and TRAIL-R2/DR5), mice encode only one, which shows modestly higher homology to TRAIL-R2 [17]. TRAIL is expressed by a wide range of hematopoietic and stromal cells, and is strongly induced by IFNs [18]. NK cells can produce high levels of TRAIL [18], [19], utilizing it as an effector molecule to kill tumor cells [20], [21]. Although TRAIL's role in regulating anti-tumor immunity is well established, its importance in anti-pathogen defense is only just emerging [22], [23]. Several studies suggest a multi-faceted role for TRAIL in antiviral immunity, where it can be both protective and pathogenic, depending upon virus- and tissue-specific factors [18], [23], [24], [25].

We have recently demonstrated that the HCMV glycoprotein UL141 can bind and suppress cell surface expression of the human TRAIL-DRs [26], expanding upon its previously known role in restricting expression of the NK cell activating ligands CD155 and CD112 [27], [28]. We now show that inhibition of TRAIL-DR signaling is conserved across the CMVs, with the m166 protein of MCMV performing a similar function as UL141 despite showing no overt sequence similarity, and is critical to thwart TRAIL-mediated NK defenses *in vivo*. M166 completely neutralizes host defenses mediated by TRAIL-DR signaling *in vivo*, with no alterations in the replication of wild-type MCMV seen in TRAIL-DR^−/−^ mice, highlighting how key insights into operable host defenses can be uncovered by studying viral immune modulatory strategies.

## Results

### M166 restricts TRAIL-DR expression in MCMV infected cells

**Figure 1:**
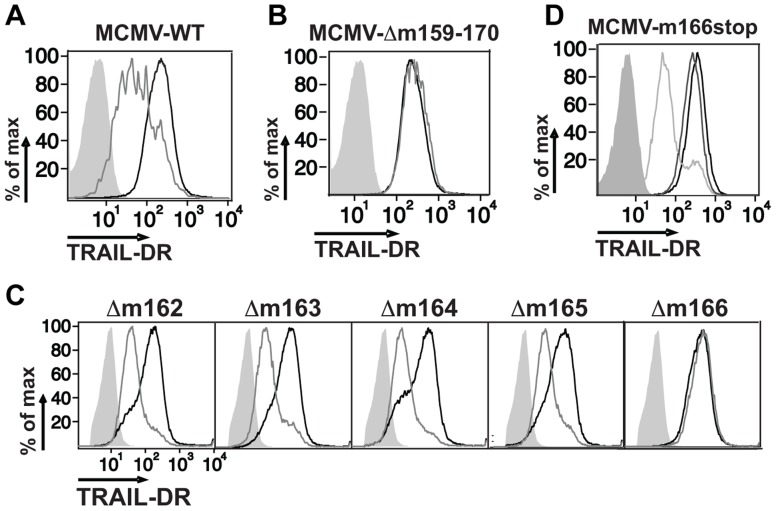
Inhibition of TRAIL-DR cell surface expression by MCMV requires m166. Fibroblasts (3T3) were mock infected or infected with WT (A), a Δm159-170 deletion mutant (B), individual orf deletion mutants (C) and a m166^stop^ mutant (D) of MCMV at a multiplicity of infection (MOI) of ∼1–2. Cell surface levels of TRAIL-DR were assessed 36–48 h later by FACS. Histograms in (A–C): black, mock; gray, MCMV; shaded, isotype control. In (D): black, mock; shaded, isotype control; light gray, WT MCMV; dark gray, m166^stop^ MCMV. All results are representative of three to four independent experiments.

CMV utilizes multiple strategies to block both extrinsic and intrinsic apoptotic pathways [29], [30], and recent data has shown that human CMV directly inhibits TRAIL-DR expression [26], [31]. To test whether mouse CMV might utilize a similar strategy, infected fibroblasts were analyzed for TRAIL-DR expression by flow cytometry (**Fig. 1A**), revealing that MCMV also inhibits cell surface levels of this DR. MCMV inactivated with UV light was unable to inhibit TRAIL-DR expression (data not shown), strongly suggesting that a virally-encoded protein performed this function. To attempt and identify the responsible MCMV open reading frame (orf), a panel of relatively large deletion mutants generated in the BAC cloned MCMV genome (Smith strain) was initially utilized [32]. TRAIL-DR surface expression was monitored after infection of 3T3 cells with these mutants, revealing a putative responsible orf(s) to be located between m159 and m170 (**Fig. 1B**). Additional smaller deletion mutants (Δm159-m161, Δm162-m166, Δ167-m170) were then utilized to identify the m162-m166 region as required for TRAIL-DR inhibition (data not shown). Subsequently, individual mutant viruses were generated disrupting these five individual orfs, revealing that an intact m166 orf appeared to be required for inhibition of TRAIL-DR cell surface expression (**Fig. 1C**).

Similar to HCMV [4], recent analysis of the MCMV transcriptome has revealed the presence of numerous, previously unrecognized transcripts arising largely from differential splicing [33]. Our initial approach inserting a kanamycin resistance cassette to identify the MCMV genomic loci that encoded the orf(s) responsible for restricting TRAIL-DR expression, although effective, has high potential to impact expression of neighboring or transcriptionally overlapping orfs. Therefore, in order to verify the predicted m166 orf-encoded protein was truly required for inhibiting TRAIL-DR expression, a stop codon was inserted in the m166 orf by ‘traceless’ mutagenesis in the BAC-cloned K181 strain of MCMV (referred to as m166^stop^ hereafter), which should be minimally disruptive to the overall locus transcription. MCMV-m166^stop^ was unable to restrict TRAIL-DR cell surface expression (**Fig. 1D**), indicating that the predicted m166 orf is required for TRAIL-DR inhibition.

### M166 expression

**Figure 2:**
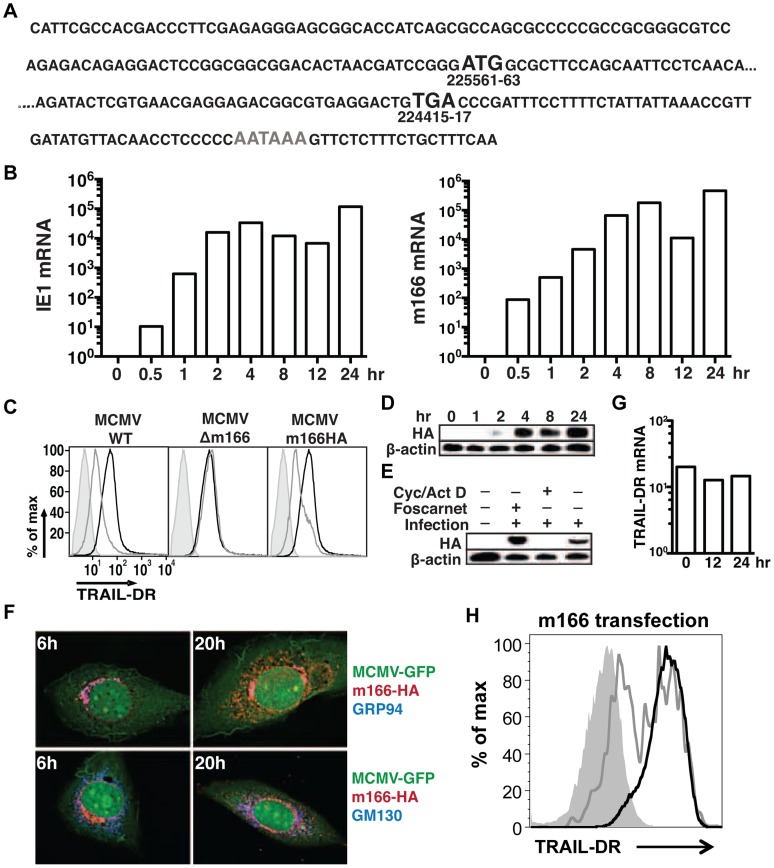
MCMV m166 expression. (A) Primary sequence of the m166 mRNA transcript as determined by RACE analysis. Black Bold: start and stop codon, gray bold: polyadenylation signal. (B) 3T3 cells were infected with MCMV-WT and expression of MCMV *m166* and *ie1* mRNA was determined by qPCR at the indicated times. All mRNA levels are normalized to L32 (×10^4^). (C) 3T3 cells were mock infected or infected with MCMV-WT, MCMV-Δm166 or MCMV-m166HA, and cell surface levels of TRAIL-DR were assessed 24 h later by FACS. Black histograms, mock; gray histograms, MCMV; shaded histograms, isotype control. (D–F) 3T3 cells were infected with MCMV-m166HA and harvested at the indicated times for Western detection of (D) m166HA protein in untreated cells, or (E) after treatment with foscarnet or actinomycin D/cycloheximide. (F) Infected 3T3 cells were co-stained with anti-HA antibody (red) along with anti-GRP94, ER marker (top panels, blue) or anti-GM130, cis-golgi marker (bottom panels, blue) antibodies at indicated times. (G) 3T3 cells were infected with MCMV-WT, and expression of TRAIL-DR mRNA was determined by qPCR at indicated times. All mRNA levels are normalized to L32 (×10^4^). All infections were performed at an MOI of ∼1. (H) 3T3 cells were transfected with an m166-GFP expression plasmid, and TRAIL-DR expression in GFP- (black histogram) and GFP+ (gray histogram) was determined by flow cytometry 72 h later. Shaded histogram, isotype control antibody.

Detailed expression analysis of m166 has not been performed to date, which is common for many of the predicted MCMV orfs. RACE analysis of transcripts emanating from the m166 locus revealed only one major mRNA initiating from the predicted start codon (**Fig. 2A**), consistent with a recently published result [33]. M166 mRNA expression was detectable by 30 minutes post infection, closely paralleling expression of the immediate early-1 (*ie1*) mRNA (**Fig. 2B**). For analysis of m166 protein expression, a recombinant MCMV was generated with a C-terminal HA epitope tag (MCMV-m166HA). MCMV-m166HA inhibited TRAIL-DR expression similarly to WT MCMV (**Fig. 2C**), and m166 protein was detectable by 2 hours post infection, robustly expressed by 4 hours and remained high at 24 hours (**Fig. 2D**). The downregulation of TRAIL-DR cell surface levels followed a similar kinetic, with reduced expression beginning at 4–8 hours and increasing by 24 hours (**Fig. S1**). The expression of m166 was inhibited by cycloheximide and actinomycin D, whereas it was not blocked by addition of foscarnet, indicating m166 is an early gene product (**Fig. 2E**). M166 was localized largely to the endoplasmic reticulum (ER) (co-localized with GRP94, ER marker) in MCMV-m166HA infected 3T3 cells at early times (6 h), while at later times (20 h) it was also detectable in the Golgi apparatus (co-localized with GM130, cis-golgi marker) (**Fig. 2F**). Endogenous TRAIL-DR could be detected in 3T3 cells only after treatment with the proteasome inhibitor MG132, but under these conditions no co-localization was observed with m166 (**Fig. S2A**). When TRAIL-DR was overexpressed, a modest level of co-localization with m166 was observed in the ER (**Fig. S2B**). At these same times when m166 was expressed largely intracellular and TRAIL-DR cell surface levels were markedly decreased, TRAIL-DR mRNA levels remained similar to uninfected cells, indicating inhibition by m166 occurs at a post-transcriptional step (**Fig. 2G**). Finally, transfection of a m166-GFP expression plasmid into 3T3 cells resulted in downregulation of TRAIL-DR cell surface expression, indicating that m166 is both necessary and sufficient to restrict its cell surface expression (**Fig. 2H**).

### M166 facilitates early replication of MCMV *in vivo*

**Figure 3:**
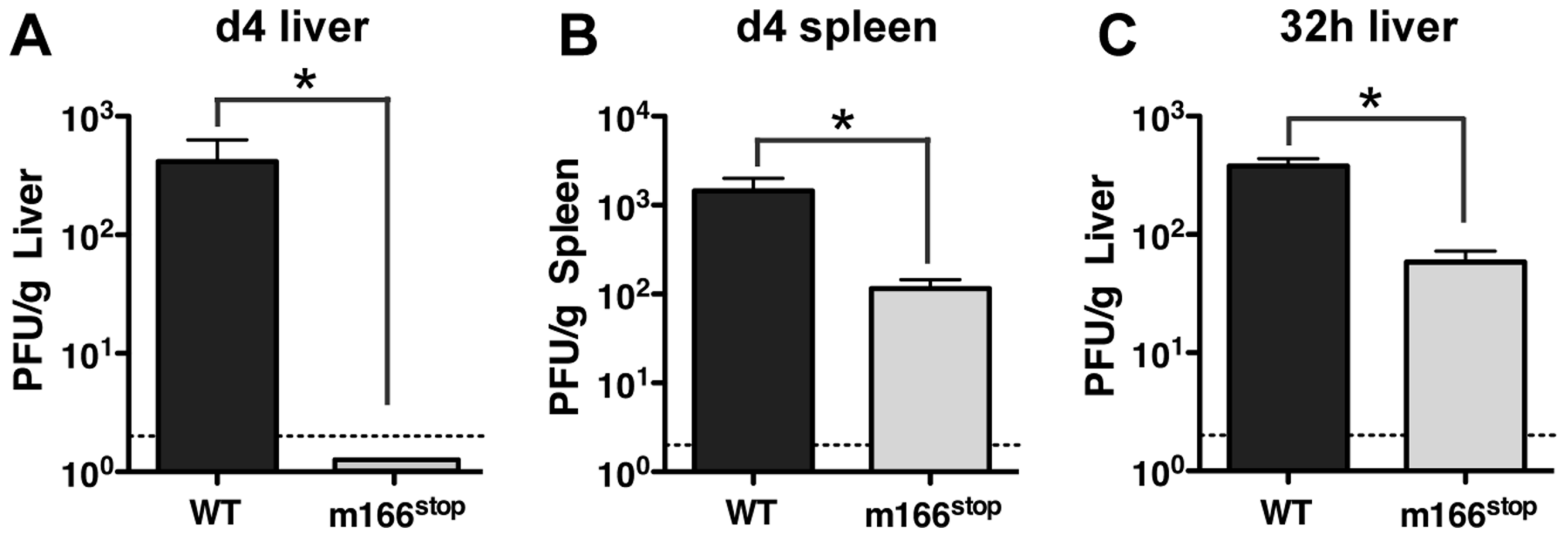
m166 is crucial for *in vivo* replication of MCMV. BALB/c mice were infected with either WT (black bars) or m166^stop^ (gray bars) MCMV and viral pfu production was measured at d4 in (A) liver and (B) spleen. Results are representative of at least four independent experiments with four mice per group. (C) MCMV pfu production in liver at 32 h after infection with WT or m166^stop^ MCMV. Results are representative of two independent experiments with at least three mice per group. Dotted line denotes the limit of assay detection. Data are represented as mean +/− SEM.

As expected, based on our observations of several Smith-based BAC mutants that lacked m166 expression, replication of MCMV K181 m166^stop^ was similar to WT in cultured fibroblasts (**Fig. S3**). However, to examine whether restriction of TRAIL-DR by m166 might contribute to *in vivo* virulence, BALB/c mice were infected with MCMV WT and m166^stop^, and replication levels were determined four days later. Mice infected with MCMV-m166^stop^ showed no detectable virus production in liver (**Fig. 3A**) and markedly reduced replication (∼15 fold) in spleen (**Fig. 3B**) with two different doses of MCMV (**Fig. S4A,B**). Significantly reduced replication of m166^stop^ was also observed in both the lung and heart at day 4 (**Fig. S4C,D**). Our recent results indicate that the ‘first burst’ of MCMV production *in vivo* occurs at ∼32 hours in the spleen and liver after systemic infection, and that innate control at this early time is dependent upon stromal cell-mediated defenses and independent of toll-like receptors [34], [35]. Interestingly, m166^stop^ replication was also compromised (∼5–10 fold) in the liver at 32 hours post infection (**Fig. 3C**). This result indicates that in addition to the absolute requirement for m166-mediated inhibition of TRAIL-DR to sustain replication of MCMV in this organ until day 4, it is also critical to thwart innate defenses at the very earliest times of infection.

### M166 inhibits NK-mediated defenses

**Figure 4:**
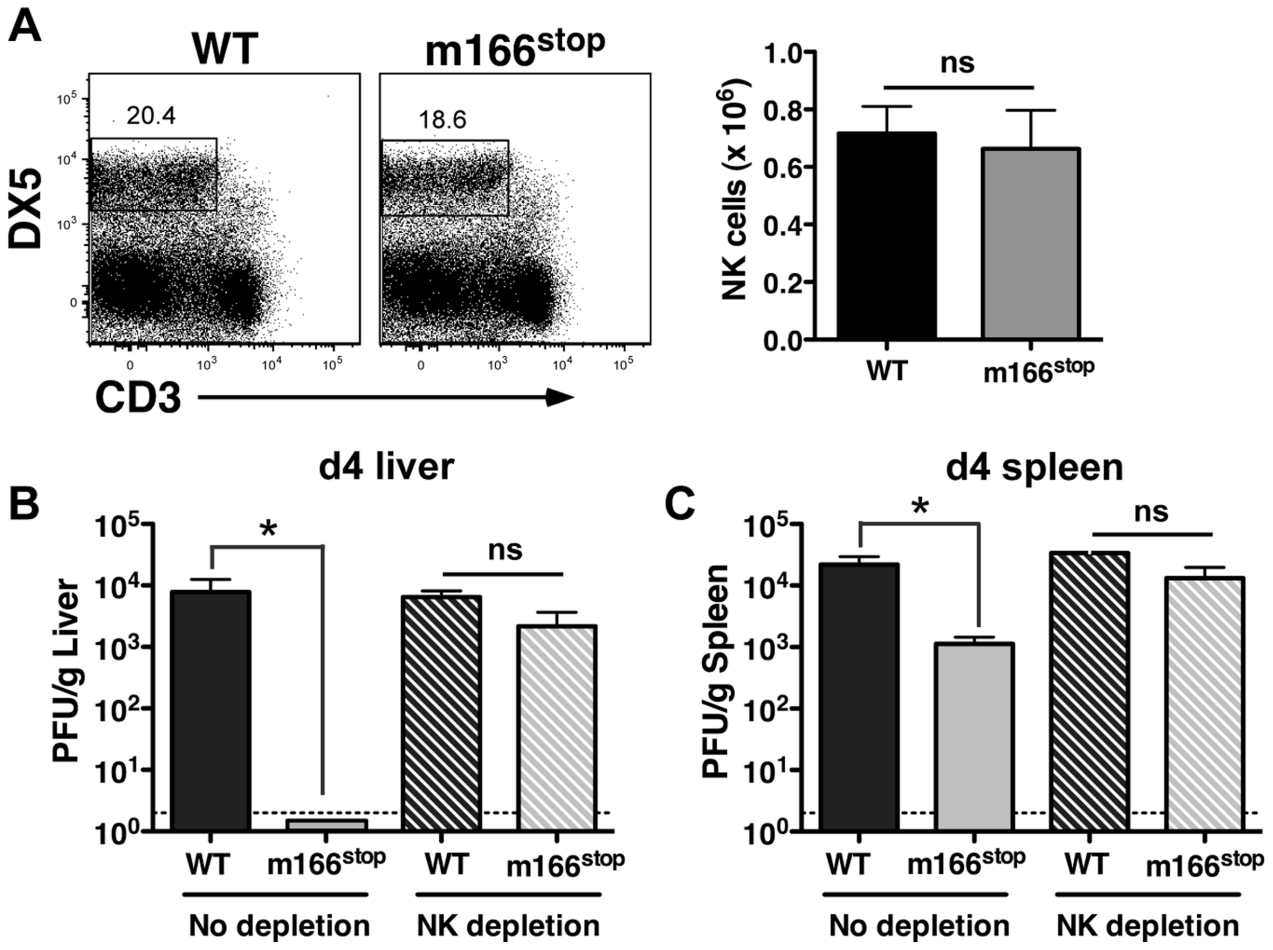
m166 inhibition of TRAIL-DR subverts NK cell antiviral defenses. (A) BALB/c mice were infected with either WT or m166^stop^ MCMV and NK cell numbers were assessed four days later. Total liver NK cell numbers after infection are shown in the bar graph. (B, C) BALB/c mice were depleted (or not) of NK cells prior to infection with WT (black bars, stippled black bars) or m166^stop^ (gray bars, stippled gray bars) MCMV. Viral pfu production was measured in liver (B) and spleen (C) 4 days later. Results are representative of 4 mice per group from a minimum of three independent experiments. Dotted line denotes the limit of assay detection. Data are represented as mean +/− SEM.

NK cells exert innate defense to MCMV infection in the spleen and liver, with the degree varying depending upon the specific inbred mouse strain [14], [36], [37]. Infection with MCMV WT and m166^stop^ resulted in equivalent numbers of resident/recruited NK cells in the liver at day 4 (**Fig. 4A**). Next, to ascertain whether MCMV-m166^stop^ was more subject to NK-mediated antiviral defenses, these cells were depleted prior to infection. NK-depletion resulted in the complete restoration of MCMV-m166^stop^ replication to WT levels in both the liver and spleen (**Fig. 4B,C**). Notably, depleting NK cells did not enhance the replication of WT MCMV in either spleen or liver of BALB/c mice (**Fig. 4B,C**), which was not entirely unexpected based on previous results [38], [39].

**Figure 5:**
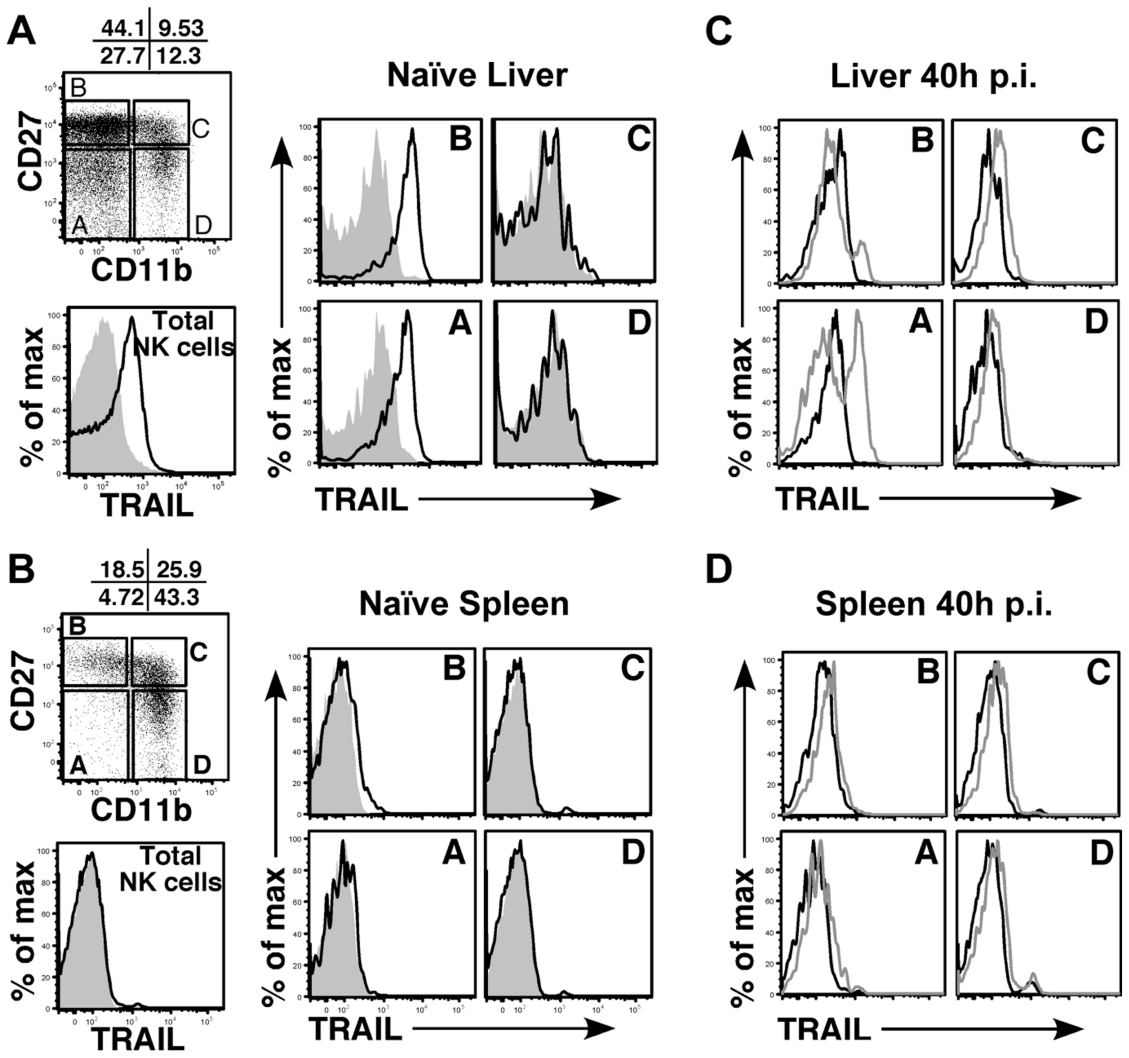
Immature liver NK cells express high TRAIL levels. Surface TRAIL expression on liver (A) and spleen (B) NK cell subsets in naïve BALB/c mice was examined by FACS. TRAIL expression on ‘total’ NK cells is shown as the black histogram. NK cells were analyzed for expression of CD27 and CD11b and subdivided into 4 maturational subsets (A–D), as shown in the dot plot. Surface TRAIL expression on the four NK subsets (black, TRAIL; gray, isotype) was analyzed. (C, D) Surface TRAIL expression was assessed on liver (C) and spleen (D) NK cell subsets 40 h after infection with WT MCMV (gray histogram). Surface TRAIL expression from naïve BALB/c mice (black histogram) is included. Results are representative of 3 mice per group from a minimum of two independent experiments.

NK cells can express TRAIL in response to virus-induced innate cytokines, so this was analyzed during MCMV infection. Tissue-resident NK cells exist in naïve, C57BL/6 (B6) mice at various stages of maturation identified by their differential expression of CD11b, DX5 and CD27, with ‘immature’ cells (CD11b^lo^) in the liver constitutively expressing cell-surface TRAIL and making up a low proportion of the total pool (<30%) [40], [41]. Similar subsets of mature and immature NK cells could be identified in naïve BALB/c mice (**Fig. S5A**), with many being CD11b^lo^ (>60%) and expressing high levels of surface TRAIL (**Fig. 5A**). TRAIL was undetectable on mature liver NK cells (CD11b^hi^), and no splenic NK cell subsets expressed detectable TRAIL in naïve mice (**Fig. 5B**). Following infection with MCMV, surface TRAIL was induced to much higher levels on approximately half of the CD11b^l^°CD27^−^ and a small percentage of CD11b^l^°CD27^+^ NK cells in the liver (5–6 fold increase in MFI in both subsets), while only very modest TRAIL induction (<2 fold) was seen on mature NK cells in this organ (**Fig. 5C**). In contrast, all NK subsets in the spleen showed very modest induction of TRAIL following MCMV infection (<2 fold over naïve), with immature subsets expressing slightly more than mature (**Fig. 5D**). In addition, TRAIL mRNA levels in FACS-purified, liver NK cell subsets paralleled its cell surface expression (**Fig. S5B**), indicating mature NK cell subsets are not likely to express high levels of secreted/soluble TRAIL compared to their immature counterparts. Importantly, NK cell numbers (**Fig. 4A**), subset proportions and TRAIL expression were not grossly different upon infection with MCMV WT and m166^stop^ (**Fig. S5C,D**), although a slight increase in the proportion of mature NK cells was observed in m166^stop^ MCMV infected livers (WT vs. m166^stop^ = 59.8% vs 68%), indicating this mutant does not induce a ‘globally different’ response. Taken together, these results show that distinct subsets of tissue resident NK cells differentially express TRAIL upon MCMV infection, and likely have distinct capacities to mediate TRAIL-dependent immune control. In addition, high levels of TRAIL expression by liver-resident NK cells correlates with the enhanced control of the m166^stop^ mutant in that organ compared to the spleen.

### M166-mediated inhibition of TRAIL-DR is critical for thwarting NK-mediated innate defense

**Figure 6:**
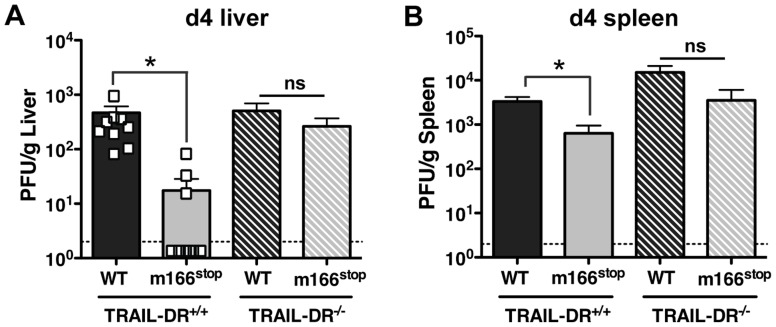
m166 inhibition of TRAIL-DR is responsible for subverting NK cell defenses. TRAIL-DR^−/−^ and littermate WT (TRAIL-DR^+/+^) mice were infected with either WT (black, stippled black bars) or m166^stop^ (gray, stippled gray bars) MCMV, and 4 days later viral pfu production was measured in (A) liver and (B) spleen. Data is composite of two separate experiments with ten mice per group. Dotted line denotes the limit of assay detection. Data are represented as mean +/− SEM.

The HCMV UL141 protein restricts expression of several cell surface molecules that regulate NK activation and/or effector function in addition to the human TRAIL-DRs (e.g. CD155 and CD112) [26], [27], [28]. Consequently, it is possible that m166 may also target additional host proteins, and was therefore critical to determine whether inhibition of TRAIL-DR expression was responsible for the attenuated replication of m166^stop^. Therefore, TRAIL-DR^−/−^ BALB/c mice were infected with MCMV WT and m166^stop^, and viral replication was measured in liver and spleen four days later. Exactly paralleling the data obtained in NK-depleted mice, m166^stop^ replication was restored to WT levels in the liver and spleen of TRAIL-DR^−/−^ mice (**Fig. 6**). Notably, no differences in the replication of WT MCMV was observed in spleens or livers of TRAIL-DR^−/−^ mice, supporting the notion that m166 completely neutralizes any potential antiviral defenses mediated by this DR *in vivo*. Naïve TRAIL-DR^−/−^ mice have similar NK subset proportions and TRAIL expression to WT BALB/c mice, and equivalent numbers of TRAIL-expressing NK cells were present in the liver of TRAIL-DR^−/−^ upon infection with both MCMV WT and m166^stop^ (**Fig. S6**). In summary, these results prove that the dominant function of m166 is to promote resistance to TRAIL-DR dependent, NK-mediated innate defense during MCMV infection.

## Discussion

Here we have identified the m166 protein of MCMV to be critical for viral inhibition of NK cell-mediated innate defense through its inhibition of TRAIL-DR cell surface expression. This is the first function ascribed to m166, and the first report that this natural mouse pathogen specifically targets this death receptor. M166 promotes early MCMV replication in an organ-specific manner, with the m166^stop^ mutant being essentially ‘dead’ in the liver while still replicating to some degree in the spleen (∼15 fold reduction). A key message from this study is that: In order to fully understand the operable host immune mechanisms activated upon infection that have antiviral potential, we must consider both the virus and host sides of the equation. Our results in TRAIL-DR^−/−^ mice show that replication of WT MCMV is unaltered, which at first glance might suggest that signaling by this TNFR has no potential to impact defenses to this β-herpesvirus. Instead, we have revealed that TRAIL-DR signaling is extremely effective at limiting MCMV replication/spread if it is not neutralized by m166 function. These results exemplify the crucial role that signaling by TNF-family cytokines plays in regulating the ‘push-and-pull’ between virus and host, exemplified by the herpesviruses, which establish lifelong persistence and employ a variety of strategies to target them.

The mouse model is commonly used to attempt and elucidate operable host immune mechanisms that may control CMV infection of humans. These two CMVs show many similarities in both their course of infection and their lasting impact on host immunity (e.g. CMV-specific T cells commonly make up >10% of the entire pool in humans and mice). However, the primary sequences of many orfs have ‘drifted’ during the >10 million years of independent evolution in their hosts [42], and it is common for immune modulatory genes encoded by primate CMVs to have no obvious sequence orthologue in their rodent counterparts. HCMV antagonizes the TRAIL/TRAIL-DR pathway through use of the UL141 glycoprotein. UL141 binds directly to human TRAIL-R2, ‘trapping’ it in the endoplasmic reticulum, reducing its cell surface levels and desensitizing cells to TRAIL-mediated killing [26]. Detection of mouse TRAIL-DR by immunofluorescence is challenging with currently available reagents. Treatment with the proteasome inhibitor MG132 induced high cellular levels and facilitated its detection in fibroblasts, and MG132 also enhances human TRAIL-R2 expression [43], but under these conditions no co-localization was observed with m166 (**Fig. S2**). Overexpression of both TRAIL-DR and m166 resulted in some ER co-localization, suggesting a potential for these two proteins to interact directly in a subcellular membrane compartment. However, we have observed no binding between purified m166:Fc and TRAIL-DR:Fc proteins (done by ELISA, data not shown), suggesting that direct binding may be low affinity, or that additional protein(s) ‘bridge’ this interaction. Additionally, m166 shows no overt primary sequence homology to UL141, and does not encode a readily identifiable Ig-domain like its functional HCMV counterpart [31]. Taken together, the data indicates that m166 and UL141 may use different mechanisms to restrict expression of TRAIL-DRs in their respective species, but suggests a strong evolutionary pressure for the convergent evolution of these two CMV genes targeting the TRAIL-DRs.

This is the first report detailing the expression and function of the MCMV m166 protein. However, previous work has suggested that m166 was likely to be important for *in vivo* fitness of MCMV [44]. In that study, the MCMV Smith strain was subjected to random insertion of an ∼3.6 kb transposon, with subsequent screening for viruses that could replicate normally in cultured cells but showed altered *in vivo* virulence. Notably, the Δm166-transposon mutant was attenuated to a higher degree in the spleen than in the liver, differing from our results. This may be ascribable to the m166^stop^ mutant having a more modest effect on expression of neighboring orfs. Additionally, the Δm166-transposon mutant was generated in the non BAC-cloned, less virulent Smith strain, which may contributes to this difference. Nevertheless, the past work validates the approach of performing random mutagenesis of the MCMV genome and screening for *in vivo* replication defects to identify gene(s) involved in subverting host immune defenses.

Several MCMV gene products (m138, m145, m152 and m155) can inhibit expression of NKG2D-activating ligands in infected cells (e.g. RAE-1, MULT-1 and H60) [39], [45], and MCMV mutants lacking these genes are compromised to varying degrees in BALB/c mice due to enhanced NK cell control. MCMV also encodes multiple proteins with the potential, or proven capacity, to block extrinsic apoptotic signaling pathways (e.g. the caspase and Bak/Bax inhibitors m36, m38.5 and m41/41.1) [30], [46]. Consequently, the m166^stop^ mutant maintains several additional, and potentially overlapping or redundant, strategies to block NK and TRAIL-dependent immune control. However, this mutant virus is dramatically attenuated *in vivo*, highlighting the critical importance of inhibiting TRAIL-DR expression for MCMV to thwart host defenses. This strongly suggests that TRAIL- and NKG2D-dependent NK effector functions operate in a mutually exclusive and non-redundant fashion in MCMV defense. Interestingly, immature human NK cells derived from cord blood preferentially utilize TRAIL-dependent killing mechanisms, as opposed to perforin/granzyme which require degranulation and involve NKG2D activation, supporting the notion that specific NK subsets utilize distinct effector mechanisms to kill target cells [47]. Perhaps because NKG2D ligands are expressed at very low levels in uninfected cells, while TRAIL-DR is expressed constitutively, m166 inhibition of TRAIL-DR has evolved to facilitate the very earliest phase of replication [35], an idea supported by our data that m166^stop^ has a lower ‘first burst’ of replication in the liver at 32 hours. Also in this vein, why is m36-mediated inhibition of caspase-8 activation, or viral inhibition of Bak/Bax, not able to compensate for the lack of m166 function? This suggests that TRAIL-induced cell death in the context of viral infection may be more complex than currently appreciated. This complexity is also likely to vary based on the infected cell type, as in the spleen where MCMV infects marginal zone stromal cells [35] the requirement for m166 is not absolute, whereas in liver hepatocytes and/or endothelial cells it is crucial. Developing robust *in vitro* assays for assessing TRAIL-mediated killing by mouse NK cells has been a challenge for the field. Our preliminary studies indicate that m166^stop^ infected L929 cells show increased caspase activation after exposure cell-expressed mTRAIL (**Fig. S7**). Experiments aimed at determining whether purified mouse NK cells operate in a similar TRAIL-dependent manner are currently underway.

Strikingly, MCMV is able to completely neutralize NK-mediated defenses in BALB/c mice. Depleting NK cells in BALB/c infected with WT MCMV did not enhance replication in the spleen or liver, with similar results being reported by the groups of Jonjic and Vidal. This is not the case in all mouse strains, most notably in B6 mice, where the MCMV m157 protein expressed on the surface of infected cells binds the Ly49H receptor, robustly activating NK cells and restricting early MCMV replication [48], [49]. Previous work with MCMV mutants unable to block NKG2D ligands revealed that the m157-Ly49H interaction ‘trumps’ the role of NKG2D inhibition in B6 mice, where these mutants show similar replication to WT virus if m157 expression is intact [45]. In general however, it is believed that the dramatic NK-dependent resistance mediated by m157-Ly49H in B6 mice is not representative of the situation in outbred mice or ‘wild’ strains of MCMV, where Ly49H is oftentimes not expressed or m157 has evolved not to bind this activating receptor [50]. Interestingly, MCMV Smith strain (non BAC-cloned) was reported to replicate to lower levels in B6 TRAIL-DR^−/−^ mice [51], something we did not observe in BALB/c mice with the K181 strain, suggesting that in the context of an extremely robust NK cell response differential roles for TRAIL-DR signaling may exist. This point is highlighted by recent data suggesting that other immune cell types expressing TRAIL, such as neutrophils, have potential to impact MCMV replication in B6 mice where NK responses are very robust [52].

CMV-induced soluble, innate cytokines also play a key role in NK activation (e.g. IFN-I, IL-12 and IL-18) [14]. IFN-I promotes TRAIL expression and NK cell cytolytic activity in both mice and humans, while IL-12/18 induces IFNγ production. The vast majority of primary human NK cells present in peripheral blood are CD56^dim^ and have no/low levels of TRAIL expression, but the ∼5% of CD56^bright^ NK cells do express TRAIL constitutively [26]. These CD56^bright^ NK are often classified as ‘immature’, perhaps paralleling the CD11b^lo^ NK subset in mice, and are present at much higher levels in tissue compared to blood [53]. Ex vivo treatment of human blood NK cells with IFN-I induces TRAIL strongly in all subsets [26], whereas in mice the CD11b^lo^ subset in the liver selectively induces TRAIL during MCMV infection. Whether these differences in the constitutive and inducible expression of TRAIL in various NK cell subsets are due to species or tissue specific reasons remains an open question. However, the fact that the m166^stop^ mutant virus is markedly more sensitive to TRAIL-DR signaling in the liver where many more NK cells express TRAIL highly, strongly suggests a key role for this extrinsic death pathway in that organ. Interestingly, TRAIL expressing CD11b^lo^ immature NK cells are present at much higher levels in fetal liver [40], and TRAIL is induced to high levels by IFN-I in HCMV infected placental cells [54], suggesting that subversion of TRAIL-DR signaling by CMV may facilitate fetal infection.

Taken together, we have shown that a viral gene product specifically inhibiting TRAIL-DR expression is critical to promote replication *in vivo*. Several other viruses restrict this pathway via direct or indirect targeting of receptor-ligand interactions and/or downstream signaling [1], but to this point few of these have been tested *in vivo*, and none have been shown to specifically promote replication by inhibiting the TRAIL-DR. Notably, as HCMV utilizes UL141 to perform a similar function as m166 [26], this strongly supports the notion that restricting the TRAIL/TRAIL-DR pathway is crucial for CMV replication in many hosts.

## Materials and Methods

### Ethics statement

This study was carried out in strict accordance with the recommendations in the Guide for the Care and Use of Laboratory Animals of the animal Welfare Act and the National Institutes of Health. All animal protocols were approved by the Institutional Animal Care and Use Committee (IACUC) of the La Jolla Institute for Allergy and Immunology, San Diego (OLAW Assurance # A3779-01).

### Cells and virus

NIH-3T3 (3T3) cells were from the ATCC (CRL1658) and 1° mouse embryo fibroblasts (MEF) were isolated from day E13.5 C57BL/6 embryos (used from passage 1–4). MEF were cultured in Dulbecco's modified Eagles medium supplemented with 10% fetal bovine serum, Pen/Strep and L-glutamine (GIBCO). 3T3 were cultured similarly, but in newborn calf serum (Omega Scientific). All cell cultures were verified to be mycoplasma free. The MCMV-GFP BAC (pSM3FR-GFP, Smith) was provided by Dr. M. Messerle. Deletions and insertions in the MCMV-GFP BAC was performed in *Escherichia coli* by ET mutagenesis and characterized as described previously [32]. WT and MCMV mutants generated on Smith BAC were used for the *in vitro* experiments performed in figures 1 and 2. The K181 MCMV-m166^stop^ mutant was generated by inserting two stop codons after the 44^th^ amino acid through ‘traceless mutagenesis’, as described [55]. The K181 BAC derived WT and m166stop mutant MCMV were used for all *in vivo* experiments shown in figures 3 through 6. Primer sequences are provided in the **Table S1**. Mutant BAC-DNAs were characterized by sequencing. MCMV virus stocks were generated by electroporating BAC DNA into 3T3 cells [56], subsequent expansion in 1° MEFs, and were quantified by standard plaque assay in 3T3 cells.

### Mice

BALB/c mice were purchased from The Jackson Laboratory (Bar Harbor, ME) and subsequently bred in house. TRAIL-DR^−/−^ B6 mice (provided by S. Schoenberger, LIAI, with kind permission from A. Winoto, UC Berkley) were backcrossed on BALB/c for 9 generations in house, and subsequently bred as heterozygotes to use +/+ and −/− littermate controls for experiments (8–12 week old, sex matched). Mice were infected intraperitoneally (i.p.) with 1×10^6^ pfu of MCMV, and pfu from infected organs were determined by plaque assay in 3T3 cells. Mice were bred under specific pathogen-free conditions in the Department of Laboratory Animal Care at the La Jolla Institute for Allergy and Immunology (LIAI), with all experiments performed in accordance with the guidelines by the Association for assessment and Accreditation of laboratory Animal Care.

### RNA isolation and analysis

Total cellular RNA was isolated at indicated time points after spin infection with TRIzol (Roche) followed by an RNeasy mini kit (Qiagen, Hilden, Germany). To assess mRNA expression in FACS sort purified NK cells from MCMV infected mice, RNA was isolated using the RNeasy micro kit (Qiagen, Hilden, Germany). Complementary DNA was generated using the iScript cDNA synthesis kit (Bio-Rad) and real-time qPCR was performed as described [57]. All mRNA levels were normalized to L32 mRNA, with primers: L32(+) 5′-ggatctggcccttgaacctt-3′, L32(−) 5′-gaaactggcggaaaccca-3′; MCMV *ie1*(+) 5′-agctgttggtggtgtcactcaa-3′, MCMV *ie1*(−) 5′-ggctgggactcatcttcttcag-3′; MCMV *m166*(+) 5′-tgcgttgggaacaactcc-3′, MCMV *m166*(−) 5′-catcgtgcactgcagacat-3′; TRAIL(+) 5′-ctaagtactcctcccttgccca-3′, TRAIL(−) 5′-tccgagtgatcccagtaatgtg-3′; CD11b(+) 5′-caatagccagcctcagtgc-3′, CD11b(−) 5′-gagcccaggggagaagtg-3′; NKG2D(+) 5′-gatggctcctctctctcatacaa-3′ and NKG2D(−) 5′-tgagccatagacagcacagg-3′.

For RACE analysis, amplification of 5′ and 3′ ends was performed using the 5′/3′ RACE kit, 2^nd^ generation (Roche) according to manufacturer's instructions. Primers: MCMV m166 5′end 5′RACEm166-2.for (5′-atgggctcgggacgcggacgc-3′), second amplification step cDNAm166-4(+) (5′-gtttgctacagtctacgagcg-3′). MCMV m166 3′end cDNAm166-4(−) (5′-cgctcgtagactgtagcaaac-3′). cDNAm166-4 (+) and (−) are reverse complementary, allowing for seamless alignment of sequencing products. The amplified products were purified by agarose gel and sequenced.

### Antibodies and flow cytometry

Anti-TRAIL (N2B2) and anti-CD27 (LG-3A10) antibodies were purchased from Biolegend; anti CD49b (pan NK, DX5), anti-CD3ε (17A2), anti CD11b (M1/70), anti-CD122 (TM-b1), anti TRAIL-R2 (MD5-1), and anti-IFNγ (XMG1.2) were purchased from eBioscience. Antibodies were conjugated to Biotin, FITC, PE, APC, PECy7, PerCP-Cy5.5, Alexa Fluor 700 and eFluor 450. To deplete NK cells, mice were injected i.p. with 50 µl of anti-asialo GM1 antibody (Wako) in 200 µl PBS 24 hours before infection. NK cell depletion was monitored in peripheral blood and spleen by FACS (>97% depletion). For detection of cell surface TRAIL-R2 levels, 3T3 cells were seeded in 6-well dishes and next day spin-infected with MCMV (1500 rpm for 5 min, turn plate and repeat). At indicated times, cells were detached with trypsin (verified not to alter TRAIL-R2 levels when compared to detachment with PBS/EDTA), washed 2× in PBS, resuspended in staining buffer (PBS +2% FCS +0.05% sodium azide) containing biotinylated anti-TRAIL-R2 antibody for 30 min on ice, washed 2× and incubated with Streptavidin-APC for 30 min (BD Pharmingen) before FACS analysis. For detection of surface TRAIL by liver NK cells, livers were perfused with PBS, minced between frosted ends of glass slides, washed and pelleted. Cell slurries were resuspended in 33.75% Percoll solution (in 1× PBS) and centrifuged at room temperature for 12 min (680 g) to pellet the mononuclear cells, washed and counted prior to use. Cells were incubated with anti-CD122, CD3, DX5, CD11b and CD27 to delineate different NK cell subsets. Liver mononuclear cells were gated for NK cells using CD122 and CD3 antibodies and further delineated into four subsets using CD27 and CD11b antibodies. TRAIL expression was detected with biotin anti-TRAIL antibody followed by streptavidin PE. Spleens were processed through a 70 µM cell strainer as described previously [35] and similarly analyzed for TRAIL expression by NK cells. Data were collected on a LSRII or FACSCalibur flow cytometer (BD Biosciences) and analyzed using FlowJo software (Tree Star). To sort purify NK cells for mRNA analysis, liver cells from MCMV infected and naïve mice were processed and stained for surface markers as described above. The four NK subsets were sorted on FACSAria and RNA isolated from sorted cells for mRNA analysis.

### Western blot

3T3 cells were infected with MCMV-m166HA. To selectively analyze immediate-early protein expression, cycloheximide (100 µg/ml; Sigma) was added to 3T3 cells 30 min prior to infection and was let sit on cells for up to 3 h post infection. At this time, cycloheximide media was washed away and actinomycin D (2.5 µg/ml; Sigma) was added to cells for another 3 h. To block late protein expression, Foscarnet (250 µg/ml; Sigma) was added just prior to infection and was present throughout. At 48 h after infection, cell pellets were scarped and lysed in protein sample buffer (3% SDS, 2% β-mercaptoethanol, 200 mM Tris [pH 8.8], 0.5M sucrose, 5 mM EDTA), boiled for 5 min, separated by SDS-PAGE (10% gel) and transferred to nitrocellulose filters. Filters were probed with anti-HA (H6908; Sigma) at 2 µg/ml followed by detection with donkey anti-rabbit HRP (GE healthcare). Signals were detected with the ECL detection kit (Amersham).

### Immunofluorescence

3T3 cells were seeded in 8 well Lab-Tek II Chambered Coverglass #1.5 Borosilicate Sterile (Nalge-Nunc) at ∼40% confluency and infected with MCMV-GFPm166HA for 6 h or 20 h. Cells were fixed in 4% paraformaldehyde/PBS for 10 min and permeabilized in PBS-0.2% Triton-X-100 for 5 min at room temperature. Rat anti-HA antibody (3F10, 1 µg/ml, Bio-Rad) plus anti-GM130 (35/GM130, 1∶300, BD Biosciences) incubations in staining buffer (1% BSA/PBS) were done overnight at 4°C, followed by incubation with Cy5 anti-rat IgG antibody (1∶300, Jackson Labs) plus PE anti-mouse IgG (1∶300, SouthernBiotech) for 1 h at room temperature. For HA + GRP94 co-stains, cells were incubated with mouse anti-HA antibody (HA.11, 1∶500, Covance) plus anti-GRP94 antibody (9G10, 1∶300, Abcam) at 4°C overnight, followed by 1 h incubation at room temperature with APC conjugated anti-mouse IgG (1∶300, Jackson Labs) plus PE anti-rat IgG (1∶300, eBioscience). Imaging was done with a Marianas inverted microscope with SlideBook software (Intelligent Imaging Innovations, Denver) (63× magnification) and analyzed using ImageJ software (National Institute of Mental Health, Bethesda, MD).

### Transfections with m166-GFP

The m166-GFP expression plasmid was generated by PCR amplification of the m166 orf from the K181 genome and cloning into the CT-GFP fusion TOPO vector (Invitrogen). 3T3 cells were seeded in a 24-well plate at 50,000 cells per well, 0.5 µg of m166-GFP plasmid DNA was incubated with 50 µL jetPRIME buffer (Polyplus) and 1.5 µL jetPRIME reagent for 10 minutes at room temperature before addition to the 3T3 cells in normal growth media. Seventy-two hours post transfection cells were analyzed by flow cytometry for TRAIL-R expression in GFP+ and GFP- cells as described.

### Statistical analysis

Statistical significance was analyzed by unpaired Student's *t* test. Unless otherwise indicated, data represent the mean ± SEM and * indicates p<0.05 and ** p<0.005.

## Supporting Information

Figure S1 **TRAIL-DR expression in MCMV infected cells.** 3T3 cells were infected with MCMV WT (K181 strain) at an MOI of 3 and analyzed for TRAIL-DR cell-surface levels by FACS at the indicated times (filled histogram, isotype; black histogram, mock infected; gray histogram, MCMV infected).
(PDF)

Figure S2 **TRAIL-DR and m166 co-localize in the ER under some experimental conditions.** (A) Immunofluorescence analysis of TRAIL-DR localization in 3T3 cells treated with MG132, with or without transient transfection of m166-GFP expression plasmid. (B) 3T3 cells transfected with TRAIL-DR alone (left panel) or in combination with m166-GFP (right panel). Analysis of the endoplasmic reticulum marker (GRP94) was is included in all panels. Expression of endogenous TRAIL-DR was undetectable in 3T3 cells without MG132 treatment.
(PDF)

Figure S3 **MCMV-m166^stop^ replicates normally in cultured fibroblasts.** 3T3 cells were infected with WT (filled squares) and m166^stop^ (open circles) MCMV (K181 strain) at an MOI of 0.03. Supernatants were collected 1, 3, 5 and 7 days later and analyzed for PFU production by plaque assay. Results are averages of three individual wells +/− SEM. The d0 time point represents the input virus PFU.
(PDF)

Figure S4 **m166 promotes MCMV replication** ***in vivo*** **.** BALB/c mice were infected with either WT (black bars) or m166^stop^ (gray bars) MCMV and replication was measured 4 days later. (A, B) Mice were infected with either 1×10^5^ (solid bars) or 1×10^6^ PFU (stippled bars) MCMV and replication was measured in (A) liver and (B) spleen. Results are representative of at least two independent experiments with four mice per group. (C, D) Mice were infected with 2×10^5^ PFU of WT and m166^stop^ MCMV and viral replication levels were assessed in (C) lung and (D) heart. Viral replication in lung represents the PFU in the entire right lobe of the lung. Dotted line denotes the limit of assay detection. Data are represented as mean +/− SEM.
(PDF)

Figure S5 **Analysis of NK cell subsets in MCMV infected mice.** (A) Liver mononuclear cells analyzed by FACS were gated for NK cells using CD3 and CD122 surface markers, which were further analyzed for CD11b and CD27 expression to identify the four NK subsets. DX5 and F4/80 surface marker expression on these four subsets was analyzed. (B) Liver NK subsets were sort purified from BALB/c mice infected with MCMV for 40 h. Expression of TRAIL, NKG2D and CD11b mRNA was assessed by qPCR. All mRNA levels are normalized to L32 (×10^5^). (C) Liver and (D) spleen NK subsets as delineated by CD27 vs CD11b surface markers and their TRAIL expression 40 h after infection with either WT (gray histogram) or m166^stop^ (black histogram) MCMV. Surface TRAIL expression from naïve BALB/c mice (shaded histogram) is included.
(PDF)

Figure S6 **TRAIL expression by NK subsets in TRAIL-DR^−/−^ mice.** (A) Naïve TRAIL-DR^−/−^ mice have similar proportions of liver NK subsets compared to WT BALB/c mice. ‘Immature’ liver NK cells from TRAIL-DR^−/−^ mice express normal levels of surface TRAIL (black, TRAIL; gray, shaded, isotype). (B) Liver NK cell numbers were assessed in TRAIL-DR^−/−^ (gray bar) and littermate WT (TRAIL-DR^+/+^, black bar) mice infected with MCMV for 4 days.
(PDF)

Figure S7 **MCMV-m166^stop^ infection sensitizes cells to TRAIL-mediated apoptosis.** TRAIL-induced apoptosis was assessed in L929 cells by measuring activated caspase 3/7 in a FACS-based assay using a FLICA probe (FLICA660-DEVD-FMK, Immunochemistry Technologies). L929 ‘targets’ were labeled with cell trace violet dye (0.5 µM, Molecular Probes) prior to seeding. Next day L929 cells were infected with WT and m166^stop^ MCMV (MOI = 5), or mock infected. Twelve hours later mock transfected (black bars) or mouse TRAIL (mTRAIL) transfected (gray bars) 293T cells were added at an E:T of 30 (+/− anti-mTRAIL blocking antibody N2B2, 20 mg/ml, white bars). Three hours later, caspase activation was measured by FACS. Results are the average of 3 individual wells +/− SEM.
(PDF)

Table S1 **Primers for construction of MCMV mutants.**
(PDF)

## References

[1] Benedict CA (2003) Viruses and the TNF-related cytokines, an evolving battle. Cytokine Growth Factor Rev 14: 349–357. 12787571 10.1016/s1359-6101(03)00030-3

[2] Lee M, Liu F (2005) Genetic analysis of cytomegalovirus by shuttle mutagenesis. Methods Mol Biol 292: 371–386. 15507721 10.1385/1-59259-848-x:371

[3] Yu D, Silva MC, Shenk T (2003) Functional map of human cytomegalovirus AD169 defined by global mutational analysis. Proc Natl Acad Sci U S A 100: 12396–12401. 14519856 10.1073/pnas.1635160100 PMC218769

[4] Stern-Ginossar N, Weisburd B, Michalski A, Le VT, Hein MY, et al (2012) Decoding human cytomegalovirus. Science 338: 1088–1093. 23180859 10.1126/science.1227919 PMC3817102

[5] Benedict CA, Crozat K, Degli-Esposti M, Dalod M (2013) Host Genetic Models in Cytomegalovirus Immunology. In: Reddehase MJ, editor. Cytomegaloviruses: From Molecular Pathogenesis to Intervention: Caister Academic Press.

[6] Cannon MJ, Schmid DS, Hyde TB (2010) Review of cytomegalovirus seroprevalence and demographic characteristics associated with infection. Rev Med Virol 20: 202–213. 20564615 10.1002/rmv.655

[7] Griffiths P, Plotkin S, Mocarski E, Pass R, Schleiss M, et al (2013) Desirability and feasibility of a vaccine against cytomegalovirus. Vaccine 31 Suppl 2: B197–203. 23598482 10.1016/j.vaccine.2012.10.074 PMC5672921

[8] Biron CA, Byron KS, Sullivan JL (1989) Severe herpesvirus infections in an adolescent without natural killer cells. N Engl J Med 320: 1731–1735. 2543925 10.1056/NEJM198906293202605

[9] Kuijpers TW, Baars PA, Dantin C, van den Burg M, van Lier RA, et al (2008) Human NK cells can control CMV infection in the absence of T cells. Blood 112: 914–915. 18650467 10.1182/blood-2008-05-157354

[10] Lopez-Verges S, Milush JM, Schwartz BS, Pando MJ, Jarjoura J, et al (2011) Expansion of a unique CD57(+)NKG2Chi natural killer cell subset during acute human cytomegalovirus infection. Proc Natl Acad Sci U S A 108: 14725–14732. 21825173 10.1073/pnas.1110900108 PMC3169160

[11] Wu Z, Sinzger C, Frascaroli G, Reichel J, Bayer C, et al (2013) Human Cytomegalovirus-Induced NKG2Chi CD57hi Natural Killer Cells Are Effectors Dependent on Humoral Antiviral Immunity. J Virol 87: 7717–7725. 23637420 10.1128/JVI.01096-13 PMC3700275

[12] Wilkinson GW, Tomasec P, Stanton RJ, Armstrong M, Prod'homme V, et al (2008) Modulation of natural killer cells by human cytomegalovirus. J Clin Virol 41: 206–212. 18069056 10.1016/j.jcv.2007.10.027 PMC2843162

[13] Babic M, Krmpotic A, Jonjic S (2011) All is fair in virus-host interactions: NK cells and cytomegalovirus. Trends Mol Med 17: 677–685. 21852192 10.1016/j.molmed.2011.07.003 PMC3205326

[14] Vidal SM, Khakoo SI, Biron CA (2011) Natural killer cell responses during viral infections: flexibility and conditioning of innate immunity by experience. Current opinion in virology 1: 497–512. 22180766 10.1016/j.coviro.2011.10.017 PMC3237675

[15] Biron CA, Nguyen KB, Pien GC, Cousens LP, Salazar-Mather TP (1999) Natural killer cells in antiviral defense: function and regulation by innate cytokines. Annu Rev Immunol 17: 189–220. 10358757 10.1146/annurev.immunol.17.1.189

[16] Ashkenazi A, Dixit VM (1999) Apoptosis control by death and decoy receptors. Curr Opin Cell Biol 11: 255–260. 10209153 10.1016/s0955-0674(99)80034-9

[17] Schneider P, Olson D, Tardivel A, Browning B, Lugovskoy A, et al (2003) Identification of a new murine tumor necrosis factor receptor locus that contains two novel murine receptors for tumor necrosis factor-related apoptosis-inducing ligand (TRAIL). J Biol Chem 278: 5444–5454. 12466268 10.1074/jbc.M210783200

[18] Sato K, Hida S, Takayanagi H, Yokochi T, Kayagaki N, et al (2001) Antiviral response by natural killer cells through TRAIL gene induction by IFN-alpha/beta. Eur J Immunol 31: 3138–3146. 11745330 10.1002/1521-4141(200111)31:11<3138::aid-immu3138>3.0.co;2-b

[19] Takeda K, Hayakawa Y, Smyth MJ, Kayagaki N, Yamaguchi N, et al (2001) Involvement of tumor necrosis factor-related apoptosis-inducing ligand in surveillance of tumor metastasis by liver natural killer cells. Nat Med 7: 94–100. 11135622 10.1038/83416

[20] Smyth MJ, Cretney E, Takeda K, Wiltrout RH, Sedger LM, et al (2001) Tumor necrosis factor-related apoptosis-inducing ligand (TRAIL) contributes to interferon gamma-dependent natural killer cell protection from tumor metastasis. The Journal of experimental medicine 193: 661–670. 11257133 10.1084/jem.193.6.661 PMC2193421

[21] Feng X, Yan J, Wang Y, Zierath JR, Nordenskjold M, et al (2010) The proteasome inhibitor bortezomib disrupts tumor necrosis factor-related apoptosis-inducing ligand (TRAIL) expression and natural killer (NK) cell killing of TRAIL receptor-positive multiple myeloma cells. Molecular immunology 47: 2388–2396. 20542572 10.1016/j.molimm.2010.05.003

[22] Benedict CA, Ware CF (2012) TRAIL: not just for tumors anymore? J Exp Med 209: 1903–1906. 23091198 10.1084/jem.20122235 PMC3478931

[23] Cummins N, Badley A (2009) The TRAIL to viral pathogenesis: the good, the bad and the ugly. Curr Mol Med 9: 495–505. 19519406 10.2174/156652409788167078 PMC3149795

[24] Dunn C, Brunetto M, Reynolds G, Christophides T, Kennedy PT, et al (2007) Cytokines induced during chronic hepatitis B virus infection promote a pathway for NK cell-mediated liver damage. J Exp Med 204: 667–680. 17353365 10.1084/jem.20061287 PMC2137916

[25] Herbeuval JP, Boasso A, Grivel JC, Hardy AW, Anderson SA, et al (2005) TNF-related apoptosis-inducing ligand (TRAIL) in HIV-1-infected patients and its in vitro production by antigen-presenting cells. Blood 105: 2458–2464. 15585654 10.1182/blood-2004-08-3058

[26] Smith W, Tomasec P, Aicheler R, Loewendorf A, Nemcovicova I, et al (2013) Human cytomegalovirus glycoprotein UL141 targets the TRAIL death receptors to thwart host innate antiviral defenses. Cell Host Microbe 13: 324–335. 23498957 10.1016/j.chom.2013.02.003 PMC3601332

[27] Prod'homme V, Sugrue DM, Stanton RJ, Nomoto A, Davies J, et al (2010) Human cytomegalovirus UL141 promotes efficient downregulation of the natural killer cell activating ligand CD112. J Gen Virol 91: 2034–2039. 20410314 10.1099/vir.0.021931-0 PMC3052539

[28] Tomasec P, Wang EC, Davison AJ, Vojtesek B, Armstrong M, et al (2005) Downregulation of natural killer cell-activating ligand CD155 by human cytomegalovirus UL141. Nat Immunol 6: 181–188. 15640804 10.1038/ni1156 PMC2844263

[29] Loewendorf A, Benedict CA (2010) Modulation of host innate and adaptive immune defenses by cytomegalovirus: timing is everything. J Intern Med 267: 483–501. 20433576 10.1111/j.1365-2796.2010.02220.x PMC2902254

[30] Mocarski ES, Upton JW, Kaiser WJ (2012) Viral infection and the evolution of caspase 8-regulated apoptotic and necrotic death pathways. Nat Rev Immunol 12: 79–88. 10.1038/nri3131 PMC4515451 22193709

[31] Nemcovicova I, Benedict CA, Zajonc DM (2013) Structure of human cytomegalovirus UL141 binding to TRAIL-R2 reveals novel, non-canonical death receptor interactions. PLoS Pathog 9: e1003224. 23555243 10.1371/journal.ppat.1003224 PMC3605307

[32] Hasan M, Krmpotic A, Ruzsics Z, Bubic I, Lenac T, et al (2005) Selective down-regulation of the NKG2D ligand H60 by mouse cytomegalovirus m155 glycoprotein. J Virol 79: 2920–2930. 15709011 10.1128/JVI.79.5.2920-2930.2005 PMC548429

[33] Juranic Lisnic V, Babic Cac M, Lisnic B, Trsan T, Mefferd A, et al (2013) Dual analysis of the murine cytomegalovirus and host cell transcriptomes reveal new aspects of the virus-host cell interface. PLoS Pathog 9: e1003611. 24086132 10.1371/journal.ppat.1003611 PMC3784481

[34] Verma S, Benedict CA (2011) Sources and signals regulating type I interferon production: lessons learned from cytomegalovirus. J Interferon Cytokine Res 31: 211–218. 21226618 10.1089/jir.2010.0118 PMC3036178

[35] Verma S, Wang Q, Chodaczek G, Benedict CA (2013) Lymphoid-tissue stromal cells coordinate innate defense to cytomegalovirus. J Virol 87: 6201–6210. 23536654 10.1128/JVI.00113-13 PMC3648091

[36] Loh J, Chu DT, O'Guin AK, Yokoyama WM, Virgin HWt (2005) Natural killer cells utilize both perforin and gamma interferon to regulate murine cytomegalovirus infection in the spleen and liver. J Virol 79: 661–667. 15596864 10.1128/JVI.79.1.661-667.2005 PMC538682

[37] Xie X, Stadnisky MD, Coats ER, Ahmed Rahim MM, Lundgren A, et al (2010) MHC class I D(k) expression in hematopoietic and nonhematopoietic cells confers natural killer cell resistance to murine cytomegalovirus. Proceedings of the National Academy of Sciences of the United States of America 107: 8754–8759. 20421478 10.1073/pnas.0913126107 PMC2889336

[38] Babic M, Pyzik M, Zafirova B, Mitrovic M, Butorac V, et al (2010) Cytomegalovirus immunoevasin reveals the physiological role of “missing self” recognition in natural killer cell dependent virus control in vivo. J Exp Med 207: 2663–2673. 21078887 10.1084/jem.20100921 PMC2989764

[39] Lodoen M, Ogasawara K, Hamerman JA, Arase H, Houchins JP, et al (2003) NKG2D-mediated natural killer cell protection against cytomegalovirus is impaired by viral gp40 modulation of retinoic acid early inducible 1 gene molecules. J Exp Med 197: 1245–1253. 12756263 10.1084/jem.20021973 PMC2193789

[40] Takeda K, Cretney E, Hayakawa Y, Ota T, Akiba H, et al (2005) TRAIL identifies immature natural killer cells in newborn mice and adult mouse liver. Blood 105: 2082–2089. 15536146 10.1182/blood-2004-08-3262

[41] Chiossone L, Chaix J, Fuseri N, Roth C, Vivier E, et al (2009) Maturation of mouse NK cells is a 4-stage developmental program. Blood 113: 5488–5496. 19234143 10.1182/blood-2008-10-187179

[42] McGeoch DJ, Dolan A, Ralph AC (2000) Toward a comprehensive phylogeny for mammalian and avian herpesviruses. J Virol 74: 10401–10406. 11044084 10.1128/jvi.74.22.10401-10406.2000 PMC110914

[43] Yoshida T, Shiraishi T, Nakata S, Horinaka M, Wakada M, et al (2005) Proteasome inhibitor MG132 induces death receptor 5 through CCAAT/enhancer-binding protein homologous protein. Cancer research 65: 5662–5667. 15994939 10.1158/0008-5472.CAN-05-0693

[44] Zhu J, Chen J, Hai R, Tong T, Xiao J, et al (2003) In vitro and in vivo characterization of a murine cytomegalovirus with a mutation at open reading frame m166. J Virol 77: 2882–2891. 12584312 10.1128/JVI.77.5.2882-2891.2003 PMC149767

[45] Jonjic S, Polic B, Krmpotic A (2008) Viral inhibitors of NKG2D ligands: friends or foes of immune surveillance? Eur J Immunol 38: 2952–2956. 18979514 10.1002/eji.200838823

[46] Handke W, Krause E, Brune W (2012) Live or let die: manipulation of cellular suicide programs by murine cytomegalovirus. Med Microbiol Immunol 201: 475–486. 22965170 10.1007/s00430-012-0264-z

[47] Zamai L, Ahmad M, Bennett IM, Azzoni L, Alnemri ES, et al (1998) Natural killer (NK) cell-mediated cytotoxicity: differential use of TRAIL and Fas ligand by immature and mature primary human NK cells. J Exp Med 188: 2375–2380. 9858524 10.1084/jem.188.12.2375 PMC2212426

[48] Arase H, Mocarski ES, Campbell AE, Hill AB, Lanier LL (2002) Direct recognition of cytomegalovirus by activating and inhibitory NK cell receptors. Science 296: 1323–1326. 11950999 10.1126/science.1070884

[49] Smith HR, Heusel JW, Mehta IK, Kim S, Dorner BG, et al (2002) Recognition of a virus-encoded ligand by a natural killer cell activation receptor. Proc Natl Acad Sci U S A 99: 8826–8831. 12060703 10.1073/pnas.092258599 PMC124383

[50] Corbett AJ, Coudert JD, Forbes CA, Scalzo AA (2011) Functional consequences of natural sequence variation of murine cytomegalovirus m157 for Ly49 receptor specificity and NK cell activation. J Immunol 186: 1713–1722. 21187440 10.4049/jimmunol.1003308

[51] Diehl GE, Yue HH, Hsieh K, Kuang AA, Ho M, et al (2004) TRAIL-R as a negative regulator of innate immune cell responses. Immunity 21: 877–889. 15589175 10.1016/j.immuni.2004.11.008

[52] Stacey MA, Marsden M, Pham NTA, Clare S, Dolton G, et al (2014) Neutrophils recruited by IL-22 in peripheral tissues function as TRAIL-dependent antiviral effectors against MCMV. Cell Host Microbe 15: 471–83. 24721575 10.1016/j.chom.2014.03.003 PMC3989063

[53] Poli A, Michel T, Theresine M, Andres E, Hentges F, et al (2009) CD56bright natural killer (NK) cells: an important NK cell subset. Immunology 126: 458–465. 19278419 10.1111/j.1365-2567.2008.03027.x PMC2673358

[54] Andrews JI, Griffith TS, Meier JL (2007) Cytomegalovirus and the role of interferon in the expression of tumor necrosis factor-related apoptosis-inducing ligand in the placenta. American journal of obstetrics and gynecology 197: 608 e601–606. 18060949 10.1016/j.ajog.2007.04.031

[55] Tischer BK, Smith GA, Osterrieder N (2010) En passant mutagenesis: a two step markerless red recombination system. Methods Mol Biol 634: 421–430. 20677001 10.1007/978-1-60761-652-8_30

[56] Messerle M, Crnkovic I, Hammerschmidt W, Ziegler H, Koszinowski UH (1997) Cloning and mutagenesis of a herpesvirus genome as an infectious bacterial artificial chromosome. Proc Natl Acad Sci U S A 94: 14759–14763. 9405686 10.1073/pnas.94.26.14759 PMC25110

[57] Schneider K, Loewendorf A, De Trez C, Fulton J, Rhode A, et al (2008) Lymphotoxin-mediated crosstalk between B cells and splenic stroma promotes the initial type I interferon response to cytomegalovirus. Cell Host Microbe 3: 67–76. 18312841 10.1016/j.chom.2007.12.008 PMC2703178

