# Supplementary figures and images for: Inhibition of the TRAIL Death Receptor by CMV Reveals Its Importance in NK Cell-Mediated Antiviral Defense
Source: PLoS Pathog. 2014 Aug 14;10(8):e1004268. doi: 10.1371/journal.ppat.1004268 (PMC4133390; doi:10.1371/journal.ppat.1004268)

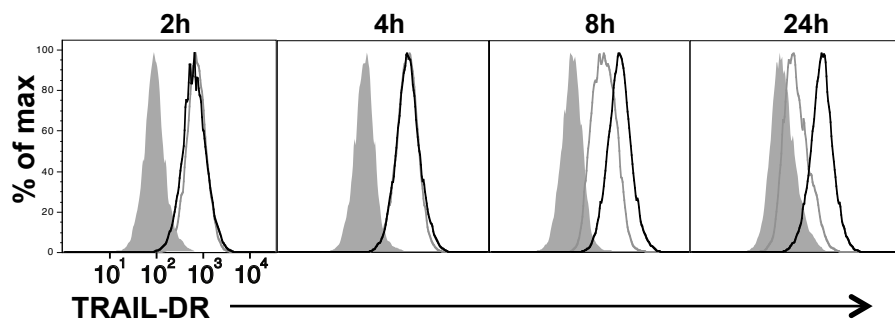

Supplement: Figure S1 — TRAIL-DR expression in MCMV infected cells. 3T3 cells were infected with MCMV WT (K181 strain) at an MOI of 3 and analyzed for TRAIL-DR cell-surface levels by FACS at the indicated times (filled histogram, isotype; black histogram, mock infected; gray histogram, MCMV infected). (PDF) [file ppat.1004268.s001.pdf]

**A**

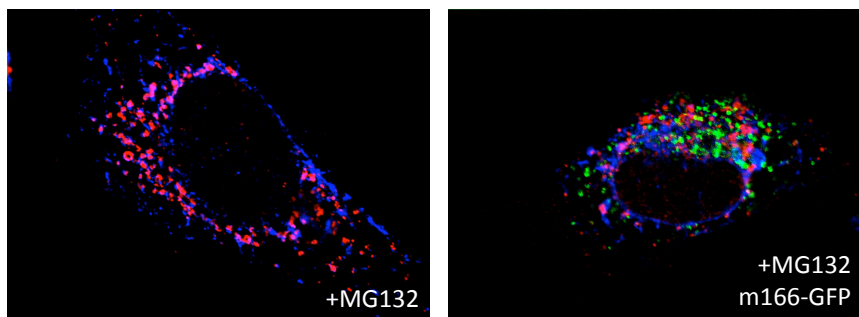

m166-GFP: green / TRAIL-DR: red / GRP94: blue

**B**

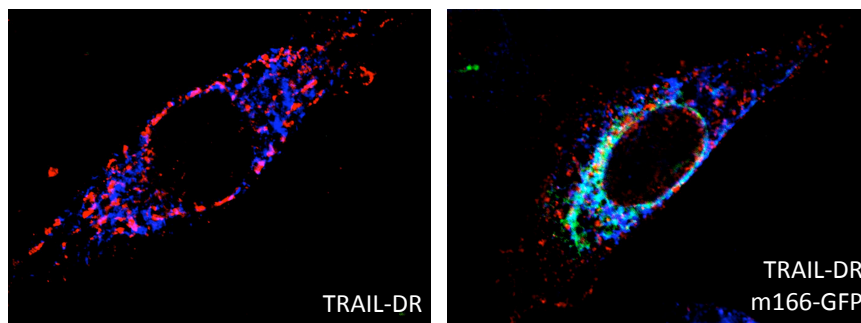

m166-GFP: green / TRAIL-DR: red / GRP94: blue

Supplement: Figure S2 — TRAIL-DR and m166 co-localize in the ER under some experimental conditions. (A) Immunofluorescence analysis of TRAIL-DR localization in 3T3 cells treated with MG132, with or without transient transfection of m166-GFP expression plasmid. (B) 3T3 cells transfected with TRAIL-DR alone (left panel) or in combination with m166-GFP (right panel). Analysis of the endoplasmic reticulum marker (GRP94) was is included in all panels. Expression of endogenous TRAIL-DR was undetectable in 3T3 cells without MG132 treatment. (PDF) [file ppat.1004268.s002.pdf]

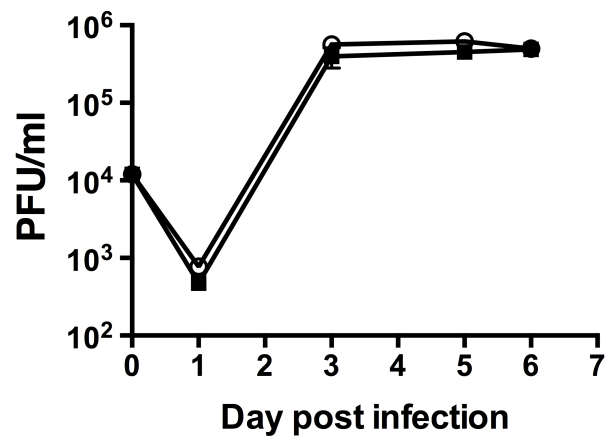

Supplement: Figure S3 — MCMV-m166stop replicates normally in cultured fibroblasts. 3T3 cells were infected with WT (filled squares) and m166stop (open circles) MCMV (K181 strain) at an MOI of 0.03. Supernatants were collected 1, 3, 5 and 7 days later and analyzed for PFU production by plaque assay. Results are averages of three individual wells +/− SEM. The d0 time point represents the input virus PFU. (PDF) [file ppat.1004268.s003.pdf]

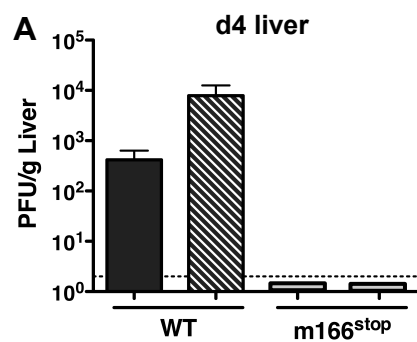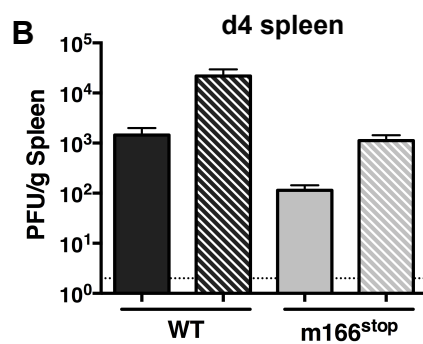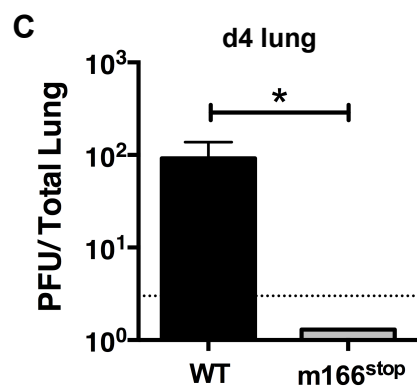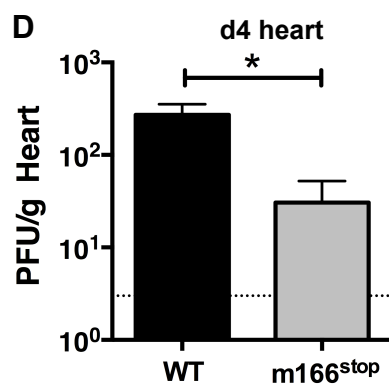

Supplement: Figure S4 — m166 promotes MCMV replication in vivo . BALB/c mice were infected with either WT (black bars) or m166stop (gray bars) MCMV and replication was measured 4 days later. (A, B) Mice were infected with either 1×10^5^ (solid bars) or 1×10^6^ PFU (stippled bars) MCMV and replication was measured in (A) liver and (B) spleen. Results are representative of at least two independent experiments with four mice per group. (C, D) Mice were infected with 2×10^5^ PFU of WT and m166stop MCMV and viral replication levels were assessed in (C) lung and (D) heart. Viral replication in lung represents the PFU in the entire right lobe of the lung. Dotted line denotes the limit of assay detection. Data are represented as mean +/− SEM. (PDF) [file ppat.1004268.s004.pdf]

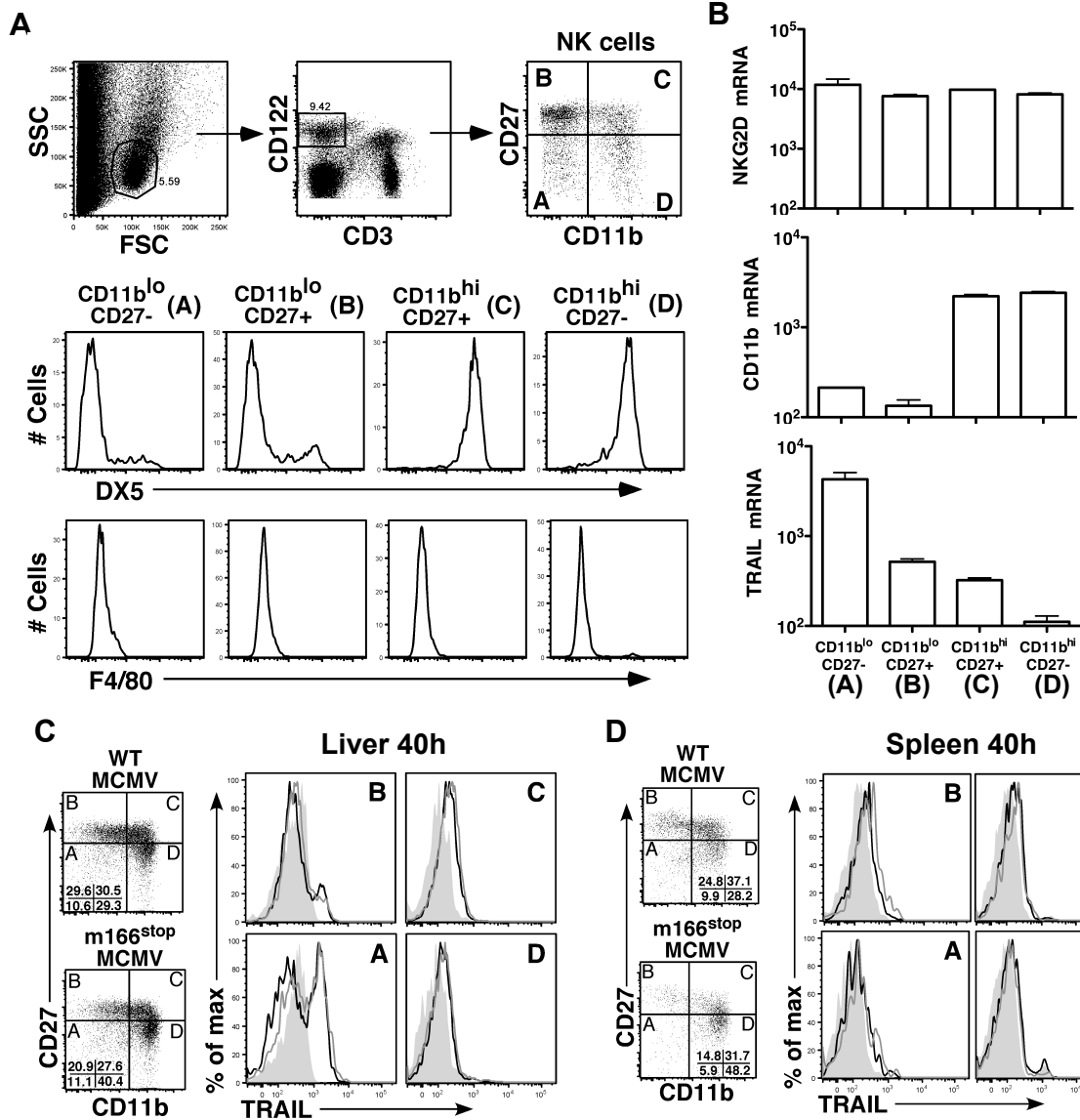

Supplement: Figure S5 — Analysis of NK cell subsets in MCMV infected mice. (A) Liver mononuclear cells analyzed by FACS were gated for NK cells using CD3 and CD122 surface markers, which were further analyzed for CD11b and CD27 expression to identify the four NK subsets. DX5 and F4/80 surface marker expression on these four subsets was analyzed. (B) Liver NK subsets were sort purified from BALB/c mice infected with MCMV for 40 h. Expression of TRAIL, NKG2D and CD11b mRNA was assessed by qPCR. All mRNA levels are normalized to L32 (×105). (C) Liver and (D) spleen NK subsets as delineated by CD27 vs CD11b surface markers and their TRAIL expression 40 h after infection with either WT (gray histogram) or m166stop (black histogram) MCMV. Surface TRAIL expression from naïve BALB/c mice (shaded histogram) is included. (PDF) [file ppat.1004268.s005.pdf]

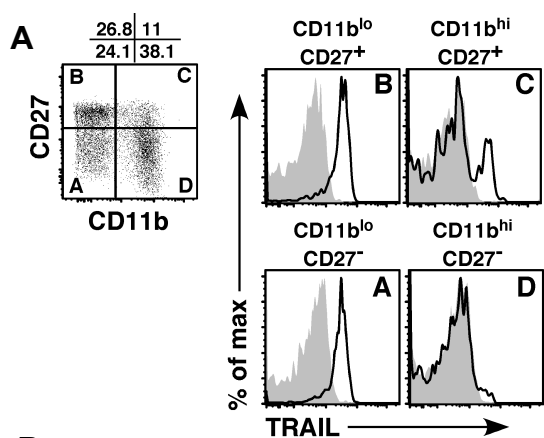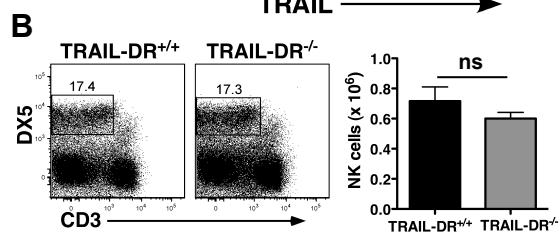

Supplement: Figure S6 — TRAIL expression by NK subsets in TRAIL-DR−/− mice. (A) Naïve TRAIL-DR−/− mice have similar proportions of liver NK subsets compared to WT BALB/c mice. ‘Immature’ liver NK cells from TRAIL-DR−/− mice express normal levels of surface TRAIL (black, TRAIL; gray, shaded, isotype). (B) Liver NK cell numbers were assessed in TRAIL-DR−/− (gray bar) and littermate WT (TRAIL-DR+/+, black bar) mice infected with MCMV for 4 days. (PDF) [file ppat.1004268.s006.pdf]

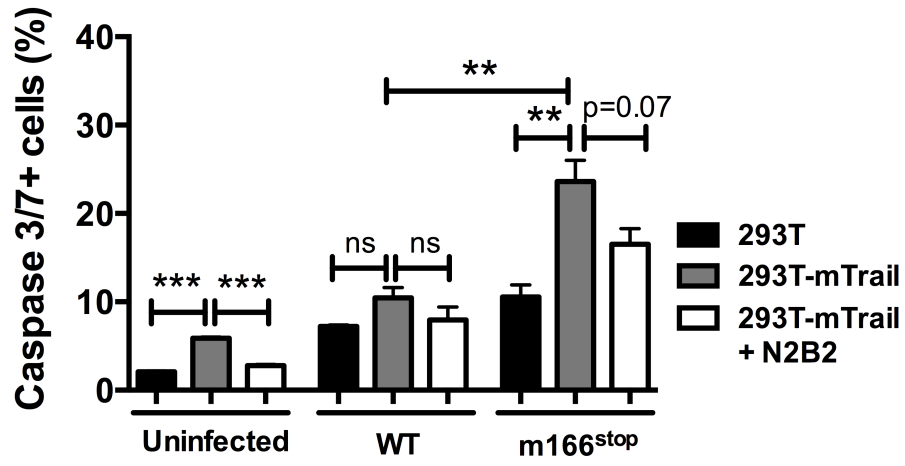

Supplement: Figure S7 — MCMV-m166stop infection sensitizes cells to TRAIL-mediated apoptosis. TRAIL-induced apoptosis was assessed in L929 cells by measuring activated caspase 3/7 in a FACS-based assay using a FLICA probe (FLICA660-DEVD-FMK, Immunochemistry Technologies). L929 ‘targets’ were labeled with cell trace violet dye (0.5 µM, Molecular Probes) prior to seeding. Next day L929 cells were infected with WT and m166stop MCMV (MOI = 5), or mock infected. Twelve hours later mock transfected (black bars) or mouse TRAIL (mTRAIL) transfected (gray bars) 293T cells were added at an E:T of 30 (+/− anti-mTRAIL blocking antibody N2B2, 20 mg/ml, white bars). Three hours later, caspase activation was measured by FACS. Results are the average of 3 individual wells +/− SEM. (PDF) [file ppat.1004268.s007.pdf]
