# Supplementary material for: Inhibition of the TRAIL Death Receptor by CMV Reveals Its Importance in NK Cell-Mediated Antiviral Defense
Source: PLoS Pathog. 2014 Aug 14;10(8):e1004268. doi: 10.1371/journal.ppat.1004268 (PMC4133390; doi:10.1371/journal.ppat.1004268)
Supplement: Table S1 — Primers for construction of MCMV mutants. (PDF) [file ppat.1004268.s008.pdf]

Table S1: Primers for construction of MCMV mutants

MCMV-GFP Δm159-170	MCMV-GFP Δm159-170.for AACTGGAAAA TATAGTTAGCA CCGTTAGAGAG GGCGACAGATTTC GATCACTACAAGG ACGACGACAATAA
	MCMV-GFP Δm159-170.rev ATGTGCTCGGT TAACGAGTTGGC CTTCGTCCGGC TGTCCGGCACG ACTACGTGAC ACAGGAACAC TTAACGGCTGA
MCMV-GFP Δm162	M162.for AGGATATGCC AGGATATGAAC CCTGCCGCCGC CACCACGGCCTC CGGTCGTCTACA AGGACGACGAC GACAAGTAA
	M162.rev ATGTATCTCT ATAATAACACC ACCTACGTCCCT GCCGACAATTAT TCCGATCACGTG ACACAGGAACAC TTAACGGCTGA
MCMV-GFP Δm163	M163.for TTTTCCGATGC TCGTGTTGAGAT CGTCGTTTATC AGAAGCGAGG TTGGGGCCTACA AGGACGACGACGA CAAGTAA
	M163.rev ACGGACCGACCA TAAGCGAAC ACCCGACGTTCA AGAGGCGCTGCAGT CCGTGACACAGGA ACACTTAACGGC TGA
MCMV-GFP Δm164	M164.for CCCGCTGCCAC GATGGCCTGGT TGTTGACGGC CCAGAAGATGC GCGAGTACCTA CAAGGACGACG ACGACAAGTAA
	M164.rev GCGAGGAGCTC CTGACGATCGAGC CGGTGGTACCGG ACGCGGCGGAGCCG TGTGACACAGGAAC ACTTAACGGCTGA
MCMV-GFP Δm165	M165.for GGCTGGAGGT AGTCCTGTCGT GCGGCGGGGACG AGAGGGAGCCGAC GAAGTCTACAAGG ACGACGACGACAA GTAA
	M165.rev CTCGCGAAGGCC GAGACCAGCGGCAG CCCAGGAGGAGACC GGGTCTCGTCAGTGA CACAGGAACACTT AACGGCTGA
MCMV-GFP Δm166	M166.for CCTCCTGGGT GACCGAGCCGAG GGCGGCGGTGGT GGTGGTTTTCTT CGGTTCTACAA GGACGACGACGA CAAGTAA
	M166.rev GCGGACACTAA CGATCCGGGAT GGCGCTTCCAGC AATTCCTCAAC AGCTATGTGACA CAGGAACACTTA ACGGCTGA

MCMV-GFP<br>m166-HA*	m166HA.for AAGGCGGAC CGGCCTATGAGA TACTCGTGAACG<br>AGGAGACGGC GTGATACC ATACGACGTCCC AGACTACGCT<br>TGAAGGACGAC GACGACAAGTAA
	m166HA.rev TCAGCATCG TAGGCCCGAGG GCCATGACGGCC<br>GGTGGCCGCG AGAAAAAGGA AGGAACACTTA ACGGCTGA
MCMV m166^{stop}	m166^{stop}.for GCTCGTCAGC CGGCCCGCGC TCGTAGACTG TAGCAAA**TAG**<br>**TAA**CCGAAGA AAACCACCAC TAGGGATAAC AGGGTAATAG<br>GATGACGACG ATAAGTAGGG
	m166^{stop}.rev GAGCCGAGGG CGGCGGCGGT GGTGGTGGTT<br>TTCTTCGGTT ACTATTTGCT ACAGTCTACG AGCGCAACCA<br>ATTAACCAAT TCTGATTAG

*Underlined: Hemagglutinin (HA) epitope sequence.
